# Novel Compound Q-1 Alleviates Type II Collagen-Induced Arthritis in Rats through the NF-*κ*B Pathway

**DOI:** 10.1155/2021/6627290

**Published:** 2021-06-30

**Authors:** Ting Xu, Jia-Chen Guo, Sha-Sha Wu, Yan Wang, Xiao-Long Liu, Hai-Bing Qian

**Affiliations:** ^1^Guizhou University of Traditional Chinese Medicine, Guiyang, China; ^2^Key Laboratory of General Higher Education Institutions in Guizhou Province, Guiyang, China; ^3^Chengdu University of Traditional Chinese Medicine, Chengdu, China

## Abstract

**Background:**

Q-1 is a novel compound extracted from the Miao medicine Tiekuaizi. Although Q-1 is known to be a coumarin derivative, its structure has not been deposited in the ACX library. Our previous study showed that Q-1 inhibits the activity of inflammatory cells. This study explores the efficacy of Q-1 in regulating rheumatoid arthritis (RA). The findings show that Q-1 acts through the NF-*κ*B signaling pathway.

**Methods:**

The effects of Q-1 were explored using a bovine type II collagen-induced arthritis (CIA) rat model. The CIA rats were intragastrically administered with high (30 mg·kg^−1^) or low (15 mg·kg^−1^) doses of Q-1. The control group was administered with an equal volume of drinking water, while the positive control group was administered with *Tripterygium* glycoside (9.45 mg·kg^−1^) for 28 consecutive days. The arthritis indices and ankle joint swelling rates were determined. The levels of IL-1*β*, IL-6, monocyte chemoattractant protein-1 (MCP-1) in serum and sialic acid (SA) in liver homogenate were determined by enzyme-linked immunosorbent assay (ELISA). The pathological features of the ankle joint were analyzed by hematoxylin and eosin (HE) staining. The I*κ*B, P-I*κ*B, P65, and P-P65 protein levels in synovial tissue were assayed by western blotting.

**Results:**

The arthritis index, ankle joint swelling rate, IL-1*β*, IL-6, and MCP-1 levels in serum, SA level in liver tissue, and I*κ*B, P-I*κ*B, P65, and P-P65 protein levels in synovial tissues were significantly higher (*P* < 0.01) in the CIA model compared to the control group. RA was successfully replicated by the CIA model, as shown by the joint swelling results and histopathological sections of the ankle. Notably, all the above indicators decreased significantly (*P* < 0.01) after treatment with Q-1 compared to the model. In addition, animals treated with Q-1 showed lower inflammation in the ankle joints than the model rats.

**Conclusion:**

The findings indicate that Q-1 effectively inhibited RA in rats by downregulating I*κ*B, P-I*κ*B, P65, and P-P65, inhibiting the excessive release of inflammatory factors, and inhibiting the overactivation of the NF-*κ*B signaling pathway.

## 1. Introduction

Rheumatoid arthritis (RA) is a chronic inflammatory synovial disease that results in damage to the body's cartilage, eventually destroying bones and joints [1]. RA is clinically manifested by joint swelling and inflammation along with the proliferation of synovial cells. Disease progression damages articular cartilage and eventually the cartilage of the limbs, leading to joint deformities [2]. Currently, drugs for RA treatment are divided into six categories: nonsteroidal anti-inflammatory drugs [3], conventional synthetic disease-modifying anti-rheumatic drugs [4], biosynthetic changes in disease anti-rheumatic drugs [5], target synthetic disease-modifying anti-rheumatic drugs [6], glucocorticoids, and herbs [7]. *Tripterygium* glycosides have been shown to have significant immunosuppressive effects [8].

In China, the current incidence of RA is approximately 0.20%–0.37%. RA causes a high rate of disability and is extremely challenging to cure [9, 10]. In the realm of Traditional Chinese Medicine (TCM), RA belongs to the “paralysis” category and is mainly caused by a deficiency in yin and yang in the internal organs and blood channels along with external cold. This leads to internal paralysis and the long-term loss of yang energy, resulting in yin deficiency [11]. Treatment approaches mainly focus on activating blood circulation, resolving stasis, clearing heat and dampness, and nourishing the liver and kidneys [12].

TCM is based on herbs with a long history of use. Many herbs show promise for the treatment of RA [13, 14]. Miao medicine is an important part of TCM. Tiekuaizi is a Miao medicine derived from the dried roots of members of the wax plum family, including *Chimonanthus praecox* (L.) Link and C. niters Oliv [15, 16]. Tiekuaizi is a common folk medicine used for treating RA in the Miao region. Previous studies have explored its chemical composition. Past studies have indicated that waxberry leaves mainly contain alkaloids, coumarins, and volatile oils. In addition, our previous studies showed that Tiekuaizi alcohol extract has an analgesic effect [17] and can effectively improve adjuvant arthritis [18, 19]. Compound Q-1, a new compound extracted from Tiekuaizi that is not found in the ACX library, has been identified as a coumarin derivative. In the present study, the anti-RA mechanism of Q-1 was explored.

## 2. Materials and Methods

### 2.1. Chemicals and Reagents

The novel compound Q-1 was isolated from Tiekuaizi in a previous study [20]. *Tripterygium* glycosides [21] (Lot 20170202) were purchased from Yuanda Pharmaceutical Huangshi Feiyun Pharmaceutical Co. (China). Purified Bovine Type II Natural Collagen (BNII) was purchased from Beijing Paix Science and Technology Co. (China). Incomplete adjuvant was purchased from Beijing Piaisi Technology Co., Ltd. (China). SA test kit (Lot 20190508) was purchased from the Nanjing Jiancheng Institute of Bioengineering (China). Enzyme-linked immunosorbent assay (ELISA) kits for IL-1*β*, IL-6, and MCP-1 (Lots R190509-113b, R190509-007b, and R190509-003b, respectively) were purchased from Simbosheng Co., Ltd. (China). I*κ*B*α*, phospho-I*κ*B*α* (Ser32), NF-*κ*B P65, and phospho-NF-*κ*B (Ser536) were obtained from Cell Signaling Technology (USA). A hematoxylin and eosin staining kit (Lot 20180608), bicinchoninic acid (BCA) protein quantification kit (Lot 20190524), 5XTRIS glycine running buffer, bovine serum albumin, and Rainbow Broad Spectrum Protein MARKER (11-180KD) were obtained from Soleibao (China). Analytical reagents were purchased from China National Pharmaceutical Group Corporation.

### 2.2. Animals and Housing

Male Wistar rats aged 6 to 8 months with weights of 180–200 g were used in this study. Animal experiments were conducted following the China Science and Technology Ministry guidelines. Animals were handled carefully to minimize injury, discomfort, and distress. The study was approved by the Animal Ethics Committee at the Guizhou University of Chinese Medicine University (Guiyang, China) and was performed in accordance to the animal use statement. Rats were housed under specific pathogen-free conditions with a 12 h/12 h dark/light cycle. All experiments were approved and performed in accordance with National Institutes of Health Guide for Care and Use of Laboratory Animals.

### 2.3. Drug Administration and Experimental Design

**Figure 1:**
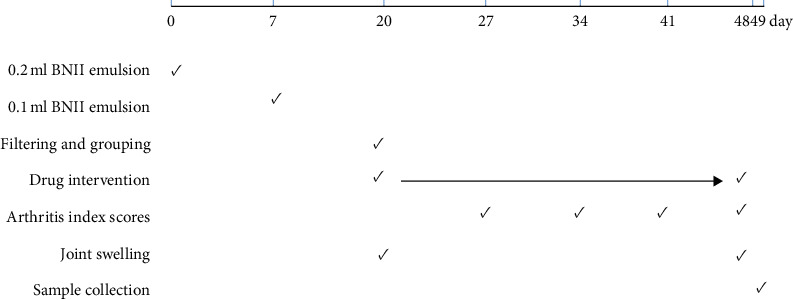
Timeline of the experimental procedure.

The CIA model was prepared following a previously described protocol [22, 23]. In summary, BNII was dissolved in 0.1 M filtered acetic acid, resulting in a BNII concentration of 1 mg/ml. CIA was induced by a single intradermal injection of 0.2 ml of the above emulsion into the base of the tail at multiple sites. One week after administration, 0.1 ml of the emulsion was injected into the same site. The day of the first immunization was defined as day 0. The clinical symptoms of the rats were evaluated every three days after the onset of clinical symptoms of CIA (around 14 days after immunization). At 20 days after immunization, the CIA rats were randomly divided into the following groups: the control group comprised non-CIA and healthy control rats; the model group comprised untreated CIA rats; the positive drug control group (PCG) was administered with 9.45 mg/kg of *Tripterygium* glycosides (according to the ratio of human and rat dose of 6.13) [24]; the Q-1 high-dose group (QHG) was treated with 30 mg/kg Q-1; and the Q-1 low-dose group (QLG) was treated with 15 mg/kg Q-1 [25]. Continuous gastric administration was performed for four weeks. Joint swelling was determined based on the paw volume, which was recorded every seven days. Arthritis indices were determined using Wood's arthritis assessment on the last day of the experiment (Figure 1).

After the experiment was completed, the rats were anesthetized via the intraperitoneal injection of urethane. Blood was collected from the abdominal aorta and centrifuged to obtain serum for the detection of IL-1*β*, IL-6, and MCP-1. The ankle joints were also collected and fixed in 10% formalin followed by staining. The animals' liver and synovial tissues were collected for SA detection and western blot analysis, respectively.

### 2.4. Analysis of Arthritis Indices

Wood's arthritis assessment criteria [26] were used to determine the RA indices at days 27, 34, 41, and 48. Ankle joint damage was assessed based on synovial hyperplasia, inflammatory cell infiltration, cartilage damage, and pannus formation using the following scale from 0 to 4 : 0 = no changes; 1 = mild; 2 = moderate; 3 = severe; and 4 = very severe. The total pathological score of ankle joint damage was determined as the sum of the individual pathological indices (resulting in a scale from 0 to 16) once a week.

### 2.5. Evaluation of the Degree of Joint Swelling

The volume under the body hair of the left ankle joint was calculated using the water dissolution replacement method based on the following formula: ankle joint swelling rate = volume measured at day 20/volume measured at day 48.

### 2.6. ELISA

Cells were lysed using RIPA lysis buffer following the manufacturer's protocol. Protein concentrations were determined using BCA protein assay. Commercial ELISA kits were used to determine the levels of IL-1*β*, IL-6, MCP-1, and SA following the manufacturer's instructions.

### 2.7. Histological Examination of Rat Knee Joints

Histological examination was performed for human synovial tissues and rat knee joints. The tissues obtained from rat knee joints were fixed with 10% formalin. The tissues were then decalcified for 20 d. The samples were embedded in paraffin, cut into 4 *μ*m sections, deparaffinized, and rehydrated for HE staining. Paraffin sections were stained with HE and analyzed.

### 2.8. Western Blot Analysis

Synovial tissues or cultured fibroblast-like synoviocytes (FLS) were treated with RIPA lysis buffer containing protease inhibitors. The supernatant was obtained, and the protein level in the lysate was determined by Bradford assay. Equal amounts of proteins (50 mg) were subjected to 10% sodium dodecyl-sulfate polyacrylamide gel electrophoresis and transferred onto polyvinylidene fluoride membranes and blocked with 5% skim milk in Tris-buffered saline. Membranes were probed with primary antibodies at 4°C overnight and incubated at 37°C with horseradish peroxidase-conjugated secondary antibodies for 2 h. Immunoreactive proteins were visualized using a Super Signal West Femto Trial Kit (Thermo Fisher Scientific, PA, USA). Protein bands were scanned and quantified by densitometry using Image J software. The relative levels of target proteins were normalized using *β*-actin as an internal control.

### 2.9. Statistical Analyses

Statistical analyses were performed using SPSS 22.0 software. Experimental data were analyzed using independent-sample *T*-test or one-way analysis of variance followed by least significant difference post hoc test. Pearson's correlation test was used to explore the correlations between the expression levels of synovial I*κ*B, P-I*κ*B, P65, and P-P65 and the pathological parameters of rat CIA or synovial *β*-actin expression. Data are presented as mean ± standard error of the mean. *P* < 0.05 was considered to indicate statistical significance.

## 3. Results

### 3.1. Q-1 Reduces the Arthritis Index in CIA Rats

**Figure 2:**
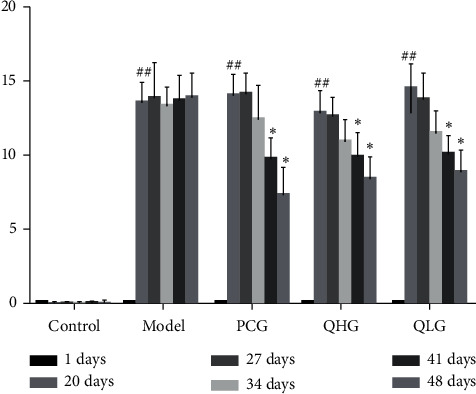
Arthritis indices in CIA rats ($\bar{x}$ ± *s*, *n* = 8). Note: ^#^*P* < 0.05, ^##^*P* < 0.01 vs. the control group; ^*∗*^*P* < 0.05, ^*∗∗*^*P* < 0.01 vs. the model group.

No statistically significant differences in arthritis index were observed between the model group and each treatment group on day 1 (Figure 2). The model group, PCG, QHG, and QLG all exhibited significantly higher arthritis indices on day 20 compared to the control group (*P* < 0.01). PCG, QHG, and QLG all exhibited significantly lower arthritis indices on day 41 compared to the model group (*P* < 0.05). These findings indicate that Q-1 can improve joint inflammation and reduce the arthritis index in CIA rats.

### 3.2. Q-1 Decreases the Rate of Ankle Joint Swelling

The model group exhibited significantly more ankle joint swelling compared to the control group (*P* < 0.01; Table 1). Ankle joint swelling was significantly attenuated in PCG, QHG, and QLG compared to the model group (*P* < 0.05 or *P* < 0.01). No significant differences in ankle joint swelling were observed between PCG, QHG, and QLG (*P* > 0.05).

### 3.3. Q-1 Downregulates Inflammatory Factors in CIA Rats

The model group, PCG, QHG, and QLG had significantly lower IL-1*β*, IL-6, and MCP-1 levels in serum and SA levels in liver tissue compared to the control group (*P* < 0.05 or *P* < 0.01; Table 2).

### 3.4. Compound Q-1 Alleviates Pathological Damage to the Ankle Joints of CIA Rats

**Figure 3:**
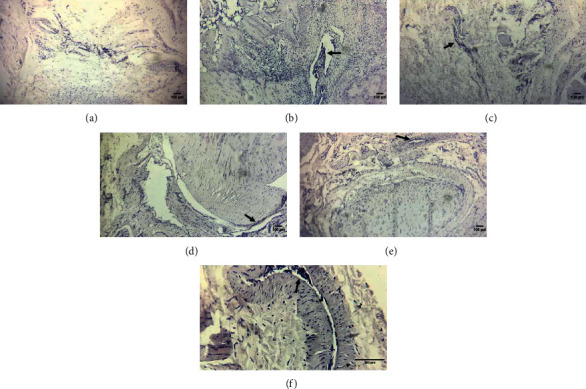
Pathological HE staining sections of the ankle joints of CIA rats: (a) control group (100×); (b) model group (100×); (c) PCG (100×); (d) QHG (100×); (e) QLG (100×); and (f) model group (40×).

No significant ankle joint damage was observed in rats in the control group (Figure 3(a)). However, rats in the model group exhibited abundant inflammatory cells (Figures 3(b) and 3(f)), indicating the successful establishment of the CIA rat model. Rats in the treatment groups exhibited significantly lower ankle joint inflammation, decreased invasion of inflammatory cells, and damage of the ankle joint compared to rats in the model group. These findings imply that Q-1 reduced changes in joint inflammation and synovial erosion in CIA rats.

### 3.5. Q-1 Downregulates I*κ*B, P-I*κ*b, P65, and P-P65 Proteins in the Synovial Tissues of CIA Rats

**Figure 4:**
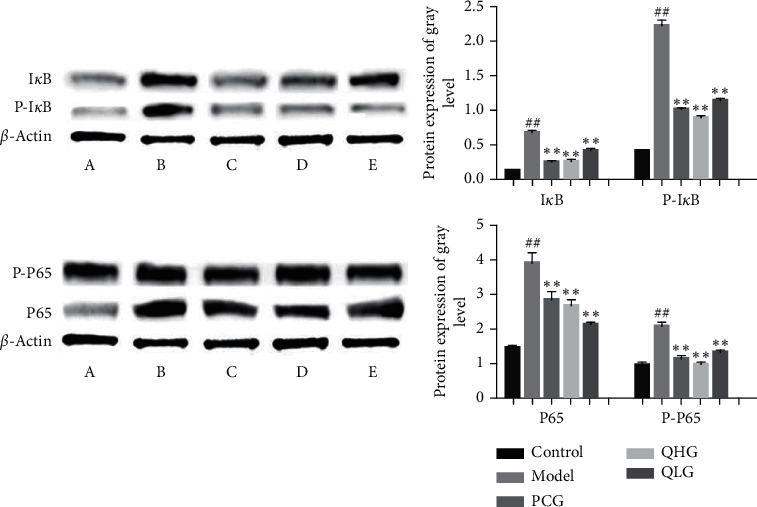
Protein expression levels of I*κ*B, P-I*κ*B, P65, and P-P65 in the synovia of CIA rats: A, control group; B, model group; C, PCG; D, QHG; and E, QLG (#*P* < 0.05, ##*P* < 0.01 vs. the control group; ^*∗*^*P* < 0.05, ^*∗∗*^*P* < 0.01 vs. the model group).

The levels of I*κ*B, P-I*κ*B, P65, and P-P65 proteins were higher in the model group than in the control group (*P* < 0.05). I*κ*B, P-I*κ*B, P65, and P-P65 protein expressions were downregulated in the synovial tissues of rats in the treatment groups compared to the model group (*P* < 0.05), indicating that Q-1 effectively inhibited the excessive activation of the NF-*κ*B signaling pathway (Figure 4).

## 4. Discussion

The clinical manifestations of RA include morning stiffness, joint swelling, pain, deformity, and dysfunction, primarily in multiple small joints of the limbs. Currently, RA pathogenesis is believed to be related to heredity, autoimmunity, and environmental factors [27]. In addition to joints, RA affects other organs such as the heart and kidneys and the respiratory system.

The NF-*κ*B signaling pathway is closely related to RA, and its activation can initiate a cascade of multiple downstream inflammatory signaling pathways to promote the secretion of inflammatory cytokines such as IL-1, IL-6, and TNF-*α*. NF-*κ*B is a classical transcription factor of inflammation downstream of TLR4, mainly composed of p65 and p50 subunits to form dimer or heterodimer, which has significant pro-inflammatory activity. At rest, the p65/p50 dimer binds to the I*κ*B inhibitor family to form a stable trimer that exists in the cytoplasm. When stimulated by extracellular signaling molecules such as cytokines and mitogens, I*κ*B dissociates from the trimer and releases p65/p50 for nuclear translocation, which specifically binds to I*κ*B sites on genes and initiates the expression of various inflammatory genes [28]. The IL family is highly unbalanced in RA cytokines, thereby accelerating the occurrence of RA. Thus, the IL family members are a key factor in inflammation. Studies have shown that IL-6 does not directly affect the formation of synovial cells and chondrocytes; however, it indirectly promotes the biological activity of IL-1, thereby inducing an inflammatory response. MCP-1 is a cytokine in the CC chemokine family. MCP-1 is a pro-inflammatory factor that plays an important role in the pathogenesis of RA. Therefore, the expression levels of MCP-1 cytokines can reflect the degree of RA inflammation. The proliferation of synovial cells can lead to the occurrence of RA and is the main cause of articular cartilage destruction and bone injury. SA is strongly associated with the occurrence of a variety of inflammatory diseases and malignant tumors; as an important part of protein glycosylation, SA is also closely related to RA [29]. Abnormal SA proliferation in RA patients aggravates the inflammatory response, making the RA more serious.

Previous studies have shown that immune inflammation caused by immune disorders is the leading cause of RA. RA activates the NF-*κ*B signaling pathway, which subsequently upregulates the levels of IL-1 and IL-6, and enhances the cellular inflammatory response. The secretion of inflammatory factors induces significant infiltration of inflammatory cells, abnormal proliferation of the synovial tissue, and the mass production of neuromuscular pannus, thereby causing joint swelling and disability [29–32].

In the present study, a CIA rat model was successfully established. The pathogenesis of CIA includes two components: humoral immunity and cellular immunity. Pathological manifestations of CIA include the proliferation of T cells and B cells in the synovial tissues of joints. In RA patients, anti-collagen antibodies appear in the serum and synovial fluid. Type II collagen induces the body to produce an autoimmune response to its tissue. This response presents as an autoantigen under RA conditions. The symptoms of the CIA rat model are similar to those of RA patients, and the CIA model is currently the best model of RA. Pathological changes in CIA model rats are similar to those of RA patients. These changes can trigger autoimmunity and are controlled by major histocompatibility complex (MHC) and related genes. Monocytes invade synovial cells and persist for long time periods. This study established a CIA rat model of RA through the collagen-induced method.

The CIA model rats showed increased levels of IL-1*β*, IL-6, MCP-1, and SA inflammation-related factors compared to the non-CIA rats. I*κ*B, P-I*κ*B, P65, and P-P65 were upregulated in the model group compared to the control group. HE staining showed that the rats' ankle joints had abundant inflammatory cells with extensive cell edema and degeneration in the model group, indicating a significant inflammatory response. Furthermore, analysis of the CIA model showed a significant activation of the NF-*κ*B signaling pathway. Q-1 treatment significantly lowered the levels of IL-1*β*, IL-6, MCP-1, and SA inflammatory factors. Moreover, Q-1 led to the downregulation of I*κ*B, P-I*κ*B, P65, and P-P65 in the synovial tissues, and significantly lower levels of inflammatory cells were observed in the rat ankle joint after Q-1 treatment. These findings indicate that Q-1 has an anti-RA effect and can reduce the levels of inflammatory factors in CIA rats. The underlying mechanism by which Q-1 inhibits ankle joint inflammation involves the downregulation of I*κ*B, P-I*κ*B, P65, and P-P65 expression and the inhibition of the excessive activation of the NF-*κ*B signaling pathway.

## 5. Conclusion

In summary, Q-1 can significantly alleviate RA in rats by downregulating the expressions of I*κ*B, P-I*κ*B, P65, and P-P65, in the synovial tissues, inhibiting the excessive release of inflammatory factors, and inhibiting the excessive activation of the NF-*κ*B signaling pathway.

## Figures and Tables

**Table 1:** Ankle joint swelling in CIA rats on day 48 ($\bar{x}$ ± *s, n* = 8).

Group	Dosage (mg·kg^−1^)	Ankle joint swelling rate (%)
Control	/	1.1 ± 0.61
Model	/	3.07 ± 1.69^##^
PCG	9.45	1.48 ± 0.49^*∗∗*^
QHG	30.0	1.44 ± 0.39^*∗∗*^
QLG	15.0	1.8 ± 0.54*∗*

Note: ^#^*P* < 0.05, ^##^*P* < 0.01 vs. the control group; ^*∗*^*P* < 0.05, ^*∗∗*^*P* < 0.01 vs. the model group.

**Table 2:** Serum IL-1, IL-6, and MCP-1 levels in CIA rats ($\bar{x}$ ± *s, n* = 8).

Group	Dosage (mg·kg^−1^)	IL-1*β* (pg/ml)	IL-6 (pg/ml)	MCP-1 (pg/ml)	SA (mg/gprot)
Control	/	171.67 ± 63.97	218.66 ± 176.93	1371.81 ± 274.92	35.51 ± 8.12
Model	/	624.64 ± 262.09^##^	795.95 ± 384.02^##^	2106.03 ± 401.25^##^	117.90 ± 21.00^##^
PCG	9.45	196.43 ± 52.20^*∗*^	260.53 ± 260.64^*∗∗*^	1546.13 ± 283.40^*∗∗*^	66.76 ± 10.93^*∗∗*^
QHG	30.0	486.24 ± 276.21^*∗∗*^	324.48 ± 261.18^*∗∗*^	1558.94 ± 243.33^*∗∗*^	79.20 ± 18.10^*∗∗*^
QLG	15.0	345.00 ± 275.04^*∗∗*^	514.34 ± 279.32^*∗*^	1814.07 ± 183.71^*∗*^	95.76 ± 17.31^*∗∗*^

Note: ^#^*P* < 0.05, ^##^*P* < 0.01 vs. the control group; ^*∗*^*P* < 0.05, ^*∗∗*^*P* < 0.01 vs. the model group.

## Data Availability

The data used to support the findings of this study are available from the corresponding author upon request.

## Supplementary Materials

Supplementary Materials This section includes the rates of ankle joint swelling in RA rats and serum IL-1*β*, IL-6, MCP-1, and SA levels in RA rats.
