# Supplementary material for: Novel Compound Q-1 Alleviates Type II Collagen-Induced Arthritis in Rats through the NF-κB Pathway
Source: Evid Based Complement Alternat Med. 2021 Jun 30;2021:6627290. doi: 10.1155/2021/6627290 (PMC8339345; doi:10.1155/2021/6627290)
Supplement: Supplementary Materials — This section includes the rates of ankle joint swelling in RA rats and serum IL-1β, IL-6, MCP-1, and SA levels in RA rats. [file 6627290.f1.docx]

**Q-1 inhibits type II collagen-induced rheumatoid arthritis in rats via the NF-κB signaling pathway**

*Xu Ting ^A B^; Guo Jia-chen ^C^; Wu Sha-sha ^A B^; Wang Yan ^A B^;Liu Xiao-long ^A B^; Qian Hai-bing ^A B^ **

*^A^ Guizhou University of Traditional Chinese Medicine, Guiyang, China*

*^B^ Key Laboratory of General Higher Education Institutions in Guizhou Province, Guiyang, China*

*^C^ Chendu University of Traditional Chinese Medicine, Guiyang, China*


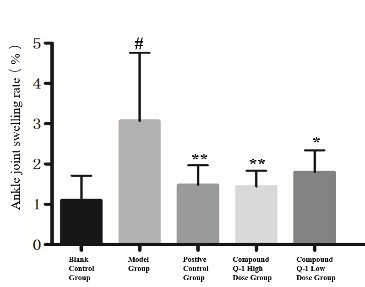


Fig.2 Rates of ankle joint swelling in RA rats (*$\overline{x}$±s，n=8*).
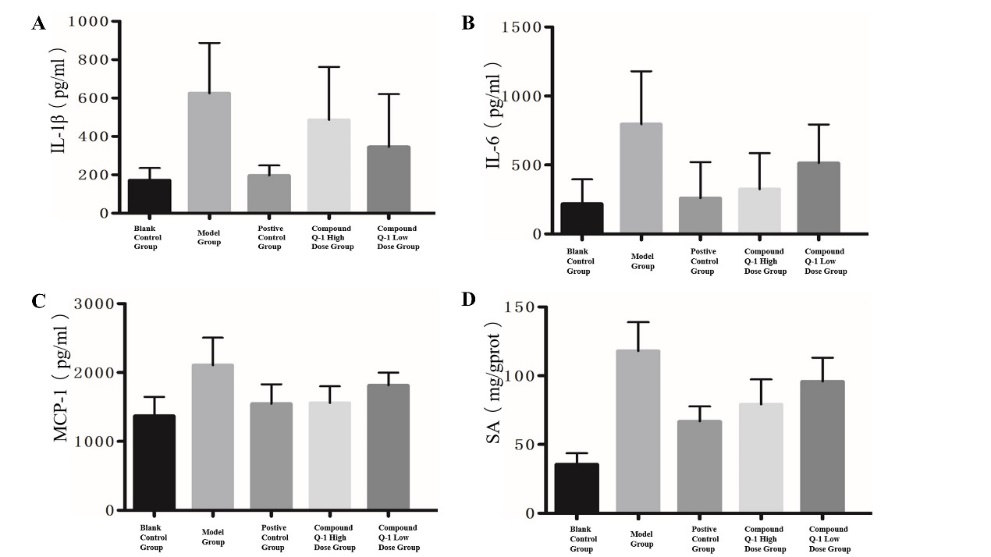


Fig. 3 Serum IL-1β, IL-6, MCP-1 and SA levels in RA rats (*$\overline{x}$±s，n=8*)
